# Sepsis modeling in mice: ligation length is a major severity factor in cecal ligation and puncture

**DOI:** 10.1186/s40635-016-0096-z

**Published:** 2016-07-18

**Authors:** Stéphanie Ruiz, Fanny Vardon-Bounes, Virginie Merlet-Dupuy, Jean-Marie Conil, Marie Buléon, Olivier Fourcade, Ivan Tack, Vincent Minville

**Affiliations:** Department of Anesthesiology and Intensive Care, Rangueil Hospital, University Hospital of Toulouse, 1 Avenue du Professeur Jean Poulhès TSA 50032, 31059 Toulouse, Cedex 9, France; Inserm/UPS UMR 1048 - I2MC, Equipe 3, Toulouse, France; EA 4564 - MATN - Laboratoire de Modélisation de l’Agression Tissulaire et de la Nociception Toulouse, Institut Louis Bugnard (IFR 150), Toulouse, France; Inserm/UPS UMR 1048 - I2MC, Equipe 12, Toulouse, France; Department of Physiology, Rangueil Hospital, University Hospital of Toulouse, 1 Avenue du Professeur Jean Poulhès TSA 50032, 31059 Toulouse Cedex 9, France

**Keywords:** Cecal ligation and puncture, Mice, Cytokine, Sepsis

## Abstract

**Background:**

The cecal ligation and puncture (CLP) model, a gold standard in sepsis research, is associated with an important variability in mortality. While the number of punctures and needle size is well described in CLP animal studies, the length of cecal ligation is often not. The relationship between cecal ligation and survival in mice is briefly reported in the literature; therefore, we devised an investigation in mice of the consequences of three standardized cecal ligation lengths on mortality and the severity of the ensued sepsis.

**Methods:**

Male C57BL/6J mice underwent standardized CLP. The cecum was ligated at 5, 20, or 100 % of its total length and further perforated by a single 20-G puncture. Mortality was analyzed. We assessed blood lactate, serum creatinine levels, and serum cytokines (TNF-α, IL-1β, IL-6, and IL-10) after procedure in a control group and in ligated mice.

**Results:**

Mortality was directly related to ligation length: median survival was 24 h for the “100 %” group and 44 h for the “20 %” group. Blood lactate increased proportionally with the ligation length. At 6 h post-procedure, pro-inflammatory cytokines significantly increased in the ligated group with significantly higher serum levels of IL-6 in the 100 % group compared to the other ligated groups. The 20 % group exhibited the characteristics of septic shock with hypotension below 65 mmHg, pro-inflammatory balance, organ dysfunction, and hyperlactatemia.

**Conclusions:**

Cecal ligation length appears to be a major limiting factor in the mouse CLP model. Thus, this experimental model should be performed with high consistency in future protocol designs.

## Background

Severe sepsis results from a complex and dynamic pathophysiology; therefore, a better understanding of the inflammatory process leading to sepsis is essential [1]. Although they do not reflect entirely the clinical complexity, animal models remain a valuable approach to developing new therapeutic strategies. Various animal models of sepsis have already been developed such as intravascular infusion of endotoxin (lipopolysaccharide (LPS)), live bacteria or viruses, bacterial peritonitis, cecal ligation and puncture (CLP), soft tissue infection, pneumonia model, and meningitidis model [2–6]. However, since 1998 Deitch pointed out that an important number of failures in new therapeutic approaches may be due to the use of inappropriate experimental models [7]. An endotoxic model (LPS injection) mimics poisoning more than infection. In the LPS endotoxic model, the cytokines peak early and transiently, whereas in the CLP model, the pro-inflammatory response is delayed and persists over time [8]. LPS model mortality occurs early, most likely due to the effects of the intense inflammatory response on the cardiovascular system, whereas in the CLP model, mortality is delayed with multiple organ failure complicating induced peritonitis. In humans, endotoxic shocks are rare and sepsis origin is often localized. The CLP model is the most widely used model for experimental sepsis and is currently considered as a gold standard in research since it mimics the nature and evolution of severe sepsis in humans [5, 9]. Ensuing a simple procedure, the model induces sepsis secondary to a stercoral peritonitis, followed by a polymicrobian translocation in the blood circulation with an early inflammatory phase, after which an anti-inflammatory response develops [2]. However, significant variability on mortality from one experimental protocol to another can lead to differing interpretations of the results. That being said, survival rates can vary from 20 to 50 %. The main determinants of mortality are the size of the needle used for cecal puncture; the number of punctures, generally between 1 and 4; and the use of antibiotics and/or fluid resuscitation [10, 11]. While the number of punctures and needle size is standardized, the length of cecal ligation is often not described in CLP animal studies.

To the best of our knowledge, only few brief descriptions exist regarding cecal ligation and survival in the mouse CLP model [10, 12]. We investigated in non-resuscitated C57BL/6J male mice the consequences of several standardized distances of CLP on mortality and sepsis severity. To do so, we used organ failure markers such as serum creatinine levels (as an early sign of acute kidney injury), serum lactate, and the kinetics of the inflammatory state reflected by cytokine synthesis including TNF-α, IL-1β, IL-6, and IL-10.

## Methods

### Animals

C57BL/6J wild-type mice were obtained from Harlan (Harlan France, Gannat, France). We used male animals aged 20 weeks, weighing 25–30 g. Animal experimentation was performed according to national and institutional animal care and ethical guidelines and was approved by the local board. Mice were housed in a temperature-controlled room on a 12-h night-dark cycle. Four animals were placed in a cage and had access to water and food ad libitum. The mice were not fasted prior to CLP procedure. The animals were shocked or control-operated and euthanized at different times after surgery.

### Protocol design

**Fig. 1:**
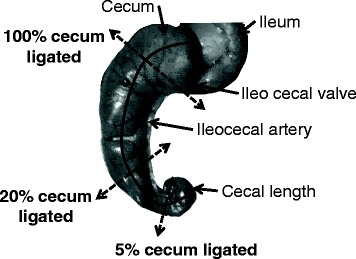
Description of cecal length ligation methods. The total length of the cecum is represented by the *full line. Dotted arrows* are placed at each level of cecal ligation: 5, 20, and 100 % of the total of cecum length

Sepsis was induced following a modification of a previously published method of CLP [10]. Briefly, animals were anesthetized with intraperitoneal injection of ketamine and xylazine (250 and 10 mg/kg, respectively). After adequate anesthesia, the lower quadrants of the abdomen were shaved and the surgical area was disinfected. A longitudinal midline incision was made using a scalpel, and scissors were used to extend the incision into the peritoneal cavity. After intramuscular, fascial, and peritoneal incision, the cecum was located and exteriorized. In our experiments, the cecum was ligated at different lengths below the ileocecal valve to avoid bowel obstruction. Total cecal length was measured from the tip of the ascending cecum to the tip of the descending cecum. The cecum was then ligated at 5, 20, and 100 % of its total length. For the “100 %” group, the cecum was ligated to the longest possible without bowel occlusion (Fig. 1).

The cecum was then perforated by a single puncture midway between the ligation and the tip of the cecum with a 20-G needle. We chose this needle diameter to obtain mid-grade lethal sepsis [5, 13, 14]. After removing the needle, a small amount of feces was extruded. The cecum was relocated, after which the fascia, abdominal musculature, and peritoneum were closed via simple running sutures; the skin was also sutured. The control mice were anesthetized and underwent laparotomy without puncture or cecal ligation and served as the control. The animals were shocked or control-operated and euthanized at different times depending on the set of experiments.

Immediately post-procedure, 1 ml of saline was administered subcutaneously for fluid resuscitation (circa 0.045 ml/g) [8, 14]. Pain control for CLP and sham mice was achieved with 0.05 mg/kg buprenorphine every 12 h.

### Spontaneous mortality determination

The first set of experiments consisted of observation of spontaneous mortality for each ligation length. The mice were then redistributed into subgroups of three to four in order to repeat the observation of mortality. The mortality was followed for 4 days after the CLP protocol.

### Renal function study and lactate dosage

In the second set of experiments, control and CLP animals were euthanized at 6 h.

We used serum lactates as a severity marker of septic state and serum creatinine concentration as a marker of renal dysfunction. Samples were collected 6 h after surgery by intracardiac puncture under general anesthesia (intraperitoneal injection of 250 mg/kg ketamine and 10 mg/kg xylazine). Serum obtained after centrifugation was immediately frozen and stored at −80 °C before being analyzed at the phenotyping platform (GenoToul Anexplo, Toulouse, France).

### Serum cytokines, bacterial blood culture, and leukocyte count

In the third set of experiments, the mice were put down at 6 and 24 h for the control, “5 %,” “20 %,” and “100 %,” ligated groups. For the control and 20 % ligated animals, we performed supplementary analysis at 48 h.

We measured serum cytokines TNF-α, IL-1β, and IL-6. IL-10 concentrations were determined at 24 and 48 h for the control and 20 % ligated groups. The samples were collected by intracardiac puncture under general anesthesia (intraperitoneal injection of 250 mg/kg ketamine and 10 mg/kg xylazine). The samples were then immediately frozen at −80 °C and analyzed by Luminex technique (Bio-Rad Y60-00000YU Pro Mouse Cytokines Group 4-plex 1 x 96, Bio-Rad, Hercules, CA, USA) on the phenotyping platform (GenoToul Anexplo, Toulouse, France).

Leukocyte count was performed at the Phenotyping platform (GenoToul Anexplo, Toulouse, France) on the MICROS-60 hematology analyzer (Horiba ABX-Diagnostics, MA, USA). Datum is expressed in leukocytes per milliliter. Analysis was performed on 20-μl samples of heparinized blood in the first hour following cardiac blood puncture.

Bacterial blood cultures were extracted for the 20 % group 24 h after CLP. The samples were collected by cardiac puncture. Blood was serially diluted and cultured on a tryptic soy blood agar plate at 37 °C for 48 h (*n* = 10 mice).

### Histological analysis

A macroscopic examination was performed to look for abscess and pus collections in the peritoneal cavity at 24 h after surgery. Liver samples, preserved in 10 % buffered formalin, were dehydrated and embedded in paraffin. Four-micrometer sections were stained with hematoxylin-eosin. The sections were then evaluated for signs of hypoperfusion and ischemic hepatitis or “shock liver.”

### Mean arterial pressure measurement

Mean arterial pressure (MAP) was measured under anesthesia in the “20 %” ligated and control groups before surgery and at 24 h post-procedure. The femoral artery was catheterized. After surgery, a 5-min stabilization period was observed and femoral arterial blood pressure was monitored using a blood pressure analyzer (via a Statham P10 EZ transducer coupled to a TA 4000; Gould, Eichstetten, Germany) for 10 min. The published results are the mean of MAP values measured every 30 s.

### Statistics

Values are not normally distributed and are expressed as median and range or interquartile range (IQR). To assess whether the measurements changed over time, Friedman’s test was used. When Friedman’s test was significant (*p* < 0.05), pair comparisons were performed using Wilcoxon’s signed-rank test. Time comparison between groups was made using non-parametric Kruskal–Wallis test. When the Kruskal–Wallis test was significant (*p* < 0.05), then comparisons were made using the Dunn’s post hoc test. Survival was analyzed by log-rank test. Analysis was performed using GraphPad Prism version 5.00 for Windows, GraphPad Software, La Jolla, CA USA, www.graphpad.com. Results with *p* < 0.05 were considered statistically significant.

## Results

### Mortality and organ dysfunctions are correlated with ligation length

**Fig. 2:**
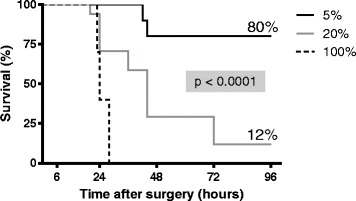
Survival following ligation of 5 % (*n* = 10), 20 % (*n* = 17), and 100 % (*n* = 10) of total cecal length within 96 h after CLP procedure. At 24 h, survival was 100 % for the 5 % group, 70 % for 20 % group, and 40 % for 100 % group (log-rank test, *p* < 0.05). At 96 h, we observed 100 % mortality in the 100 % ligated group, 88 % mortality in the 20 % ligated group, and 20 % mortality in the 5 % ligated group (log-rank test, *p* < 0.0001). The median survival time was 24 h for the 100 % ligated group and 44 h for the 20 % group. The median survival of the 5 % ligated group could not be determined because of the low number of deaths at the end of the observation period (80 % of survival)

**Fig. 3:**
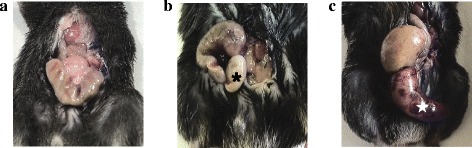
Macroscopic aspects of ceca 24 h after CLP procedure. **a** Sham-operated mouse. **b** Mouse with 20 % of cecum ligated; we observed cecal abscess (). **c** Mouse with cecum ligated at 100 % presented an ischemia of the ligated part ()

Median total cecal length was 29 mm (25–30 mm). For the ligated groups, measurements of ligated ceca were the following: 2 mm (1–2 mm) for the 5 % ligated group, 6 mm (5–7 mm) for the 20 % ligated group, and 21 mm (19–25 mm) for the 100 % ligated group. Mortality was evaluated at different ligation lengths of standardized CLP by simple puncture with a 20-G needle. Our results indicate that the ligation length influences mortality (Fig. 2). At the end of the 96-h follow-up period, we observed 100 % mortality in the 100 % ligated group, 88 % mortality in the 20 % ligated group, and 20 % mortality in the 5 % ligated group (*p* < 0.001). The median survival time was 24 h for the 100 % ligated group and 44 h for the 20 % group. The median survival time of the 5 % ligated group could not be determined because of the low number of deaths at the end of the observation period (80 % of survival). Because of death rapidity in the 100 % group, we macroscopically analyzed ceca 24 h post-procedure when the animals were put down for serum collection. The mice with cecum ligated at 100 % presented with ischemia of the ligated component in contrast to other groups (Fig. 3c). Animals in the 20 % group developed macroscopic cecal abscesses (Fig. 3a, b).

**Fig. 4:**
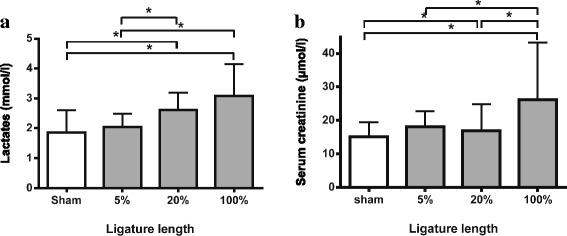
Serum creatinine and blood lactate variations according to ligation distance at 6 h after procedure as markers of organ dysfunction. **a** Blood lactate increased proportionally with the length of the ligated cecum. **b** At 6 h, serum creatinine augmented significantly 1.5 times in the 100 % group compared to the sham mice. Results are reported as median ± IQR. *p < 0.05; *n* = 14 to 20 per group

Serum creatinine and blood lactate also varied with ligation distance. Blood lactate increased proportionally with the length of ligated cecum (Fig. 4a). For the 5 % ligated group, it did not differ from the control group. For the 20 % and 100 % ligated groups, blood lactate increased up to 2.6 mmol/l (1.2–4.2) for the 20 % group and up to 3.2 mmol/l (1.2–5.7) for the 100 % group. At 6 h, serum creatinine increased by 1.5-fold in the 100 % group compared to the control mice: 26.2 μmol/l (17.4–73.8) vs 15.2 μmol/l (6.6–23.9). In the 20 % group, serum creatinine was higher at 16.9 μmol/l (12.5–39) when compared to the control group (Fig. 4b).

### Inflammatory status differs in accordance with ligation length

Inflammatory response to CLP length was evaluated by serum cytokines. TNF-α, IL-1β, and IL-6 concentrations were measured at 6 and 24 h for all the groups. We calculated an IL-6/IL-10 ratio at 24 h to determine the balance between pro- and anti-inflammatory responses. At 24 h post-procedure, only four animals in the 100 % group were still alive.

**Fig. 5:**
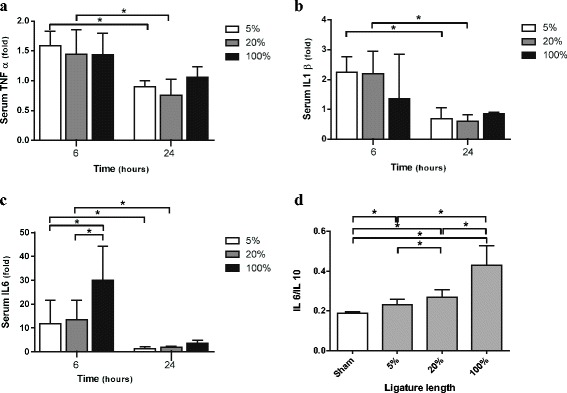
Inflammatory response to CLP length evaluated by serum cytokines. TNF-α, IL-1β, and IL-6 concentrations were measured for all the groups at 6 h (*n* = 10 for sham-operated and each length of the ligated cecum) and 24 h (*n* = 10 for the sham-operated, 5 %, and 20 %; *n* = 4 for the 100 % ligated group). At 24 h after procedure, only 40 % of the animals in the 100 % group were alive. We expressed serum TNF-α, IL-1β, and IL-6 concentrations as fold increases relative to time-matched sham laparotomy (**a**–**c**). **a** Serum TNF-α concentrations. We did not observe any difference between the ligated groups at H6 or H24, but a decrease in time. **b** Serum IL-1β concentrations. We observed a decrease in time, but no difference between the ligated groups at the same time. **c** Serum IL-6 concentrations. Besides decrease in time, we observed significant higher levels of serum IL-6 at H6 in the 100 % compared to the 5 % and 20 % ligated groups. **d** Serum IL-6/IL-10 ratio. The longer the ligated cecum was, the more the imbalance was significantly in favor of pro-inflammatory response. Results are reported as median ± IQR; *p < 0.05

At 6 h after the procedure, pro-inflammatory cytokines were significantly increased in the ligated groups compared to the control group. TNF-α was increased in the 5 % group (212.4 pg/ml (56.7–313.7)), 20 % (187.6 pg/ml (105.6–317)), and 100 % group (190.9 pg/ml (112.1–317.1)) compared to the control group (136.7 pg/ml (50.3–181); *p* < 0.05). We did not observe any difference in amongst the ligated groups (Fig. 5a). At 24 h, TNF-α quickly decreased or animals subsequently died. There was no difference between the 100 % groups because of the few number of survivors at 24 h (less than five) (Fig. 5a).

IL-1β significantly increased at 6 h for the groups 5 % (218.8 pg/ml (26–320.8)) and 20 % (213.4 pg/ml (119–388.6)) compared to the control group (93.5 pg/ml (67.5–150.1); *p* < 0.05). On the other hand, IL-1β serum concentrations of the 100 % ligated group were not different from the control group (128.4 pg/ml (60.8–354.9)). IL-1β concentrations did not increase in accordance with the length of ligation at 6 h but decreased at 24 h nevertheless (Fig. 5b).

At 6 h, IL-6 serum concentrations of ligated groups were at least 15 times higher than in the control group (Fig. 5c; *p* < 0.05). Serum IL-6 concentrations reached 1916 pg/ml (961–4141) for the 20 % ligated group and were evidently increased for the 100 % group, with a median value of 4262 pg/ml (2070–7723). Like other pro-inflammatory cytokines, IL-6 concentrations decreased at 24 h (Fig. 5c).

At 24 h, when observing the pro- and anti-inflammatory balance (IL-6/IL-10), the longer the ligated cecum, the more significant the pro-inflammatory status was (Fig. 5d).

### The “20 %” ligated group presented all characteristics of septic shock

**Fig. 6:**
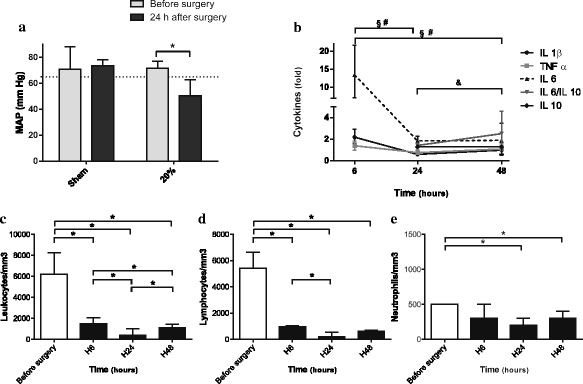
Clinical and biological characteristics of the 20 % ligated group. Results are reported as median ± IQR. **a** Invasive mean arterial pressures (MAP) were measured in the 20 % ligated and sham groups before surgery and at 24 h post-procedure. At 24 h after surgery, the animals presented a decreased MAP below 65 mmHg compared to sham mice. *p < 0.05. **b** TNF-α, IL-1β, IL-6, IL-10, and IL-6/IL-10 ratio serum concentrations were measured at 6 h (H6), 24 h (H24), and 48 h (H48) after surgery. We expressed serum cytokine concentrations as fold increases relative to time-matched sham laparotomy (*n* = 10 for the sham-operated and 20 % group at H6, H24, and H48). We observed a significant imbalance in favor of pro-inflammatory status. ^§^ *p* < 0.05 for IL-1β between H6 and H24, and H6 and H48 after CLP. ^#^ *p* < 0.05 for TNF-α between H6 and H24 and H6 and H48 after CLP. &*p* < 0.05 for IL-6/IL-10 between H24 and H48. **c, d** Leukocyte (**c**), lymphocyte (**d**), and neutrophil (**e**) counts in the 20 % ligated group before surgery, at H6, H24, and H48. We observed a drop after surgery, more pronounced at H24, with an increase at 48 h. *p < 0.05

With these results, we more closely monitored the 20 % ligated group. At 24 h after surgery, without any resuscitation the animals presented with a decreased MAP below 65 mmHg compared to the control mice (Fig. 6a).

**Fig. 7:**
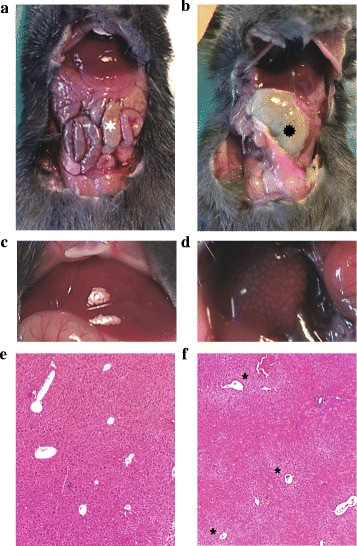
Aspect of peritoneal cavity of the sham-operated mice (**a, c, e**) and the 20 % ligated mice (**b**, **d**, **f**) 24 h after surgery. **a** The cecum is identified by a *white star*. **b** The 20 % ligated animal with cecal abscess (). When observing liver macroscopic morphology, we found a patchy appearance corresponding to pale ischemic areas (**d**) in contrast to the sham liver (**c**). **f** These areas were centrilobular necrosis of hepatocytes (*asterisk*) (**e** and **f** hematoxylin-eosin coloration ×50)

This hypotension was associated with sepsis in the 20 % ligated group. We observed cecal abscesses when compared to control mice (Fig. 7a, b), and blood cultures at 24-h post-procedure were positive with enteric bacteria such *Citrobacter braakii* and *Enterococcus faecalis* (40 %).

This was associated with a drop in leukocyte count due to leucopenia, which peaked at 24 h (Fig. 6c, d, e). At 48 h, leukocyte count increased but was diminished compared to time before surgery. The cytokine profile was pro-inflammatory, including status at 48 h. IL-6 levels remained high in survivors at 2.9 compared to those in the control and were not counterbalanced by IL-10 levels. At 48 h, IL-6/IL-10 ratio increased compared to 24 h after CLP, what highlights the persistence of inflammation (Fig. 6c).

When observing macroscopic liver morphology, we found a patchy appearance corresponding to pale ischemic areas in contrast to the control liver (Fig. 7c, d). These areas displayed centrilobular necrosis of hepatocytes (Fig. 7f).

## Discussion

While the CLP model is the most widely used model for experimental sepsis, the length of cecal ligation is often inaccurately described. Our results underline that, in mice, length of cecal ligation is a major determinant of mortality and sepsis severity. Organ dysfunction markers and pro-inflammatory status increased with ligation length. The “20 %” ligated group presented all characteristics of septic shock with a delayed mortality compared to the “100 %” group, allowing further studies as to the effect of different treatments or physiopathology. In the “20 %” group, we confirmed the presence of sepsis associated with hypotension below 65 mmHg, pro-inflammatory balance with high IL-6 levels and augmented IL-6/IL-10 ratio, organ dysfunction, hyperlactatemia, elevated serum creatinine, and hepatocyte centrilobular necrosis.

Length of ligated cecum influenced mortality in our mice model with high lethality in the 20 % and 100 % groups (at 4 days, 88 and 100 % mortality, respectively). As we found in our results, the percentage of cecum ligation is more accurate than a standard length (as length can vary from 25 to 30 mm). Rittirsch briefly described this influence in his mouse CLP procedure but did not characterize the model except for survival rate and for pro-inflammatory cytokines in his mid-grade sepsis group [10, 12]. Singleton showed the same influence more extensively but in a rat CLP model [15]. In his model, at 96 h a ligation length of 20 % allowed a 60 % survival rate whereas “25 %” of a ligated cecum caused higher mortality with only a 24 % survival rate. Our data confirm the importance of this variable in mouse CLP model. Singleton’s results could not be transposed a priori because of differences in anatomy and vascularization of these rodents’ ceca. In fact, the same proportion of ligated cecum between these two breeds of animals does not exactly have same consequence in terms of lethality. Our study was not designed to determine which mechanisms were involved in this phenomenon. However, we suppose that mortality in the 100 % ligated group is related to intestinal ischemia rather than sepsis. We observed macroscopic cecal necrosis early after procedure. As discussed in Singleton’s study concerning rats, the response amplitude to cecum ligated length may be due to the amount of feces stored in the ligated portion and thus create bacterial inoculum that may translocate or be locally pathogenic (e.g., by forming abscesses like in the 20 % group).

Inflammatory response mediated by cytokines plays a major role in sepsis evolution [16, 17]. Pro-inflammatory cytokines like TNF-α, IL-1β, and IL-6 are responsible for severe manifestations in sepsis and septic shock [17, 18]. In our model, apart from IL-6 measurements, differences between groups for other cytokines are not clinically relevant when considering the same measurement time. Serum TNF-α levels peak at 120 min in mouse CLP models [19]. It is the first cytokine produced in response to aggression, and it promotes the activation of immune cells and the release of immunoregulatory mediators [20]. In our model, TNF-α was significantly increased at 6 h in ligated groups compared to control mice. However, serum TNF-α concentrations were not proportional to ligation length as described previously in rats [15]. We detected serum TNF-α in the control mice as well and did not find any differences between the ligated groups; this finding was opposite to Singleton et al.’s in rats [15]. These findings are in accordance with other studies observing severity in mice CLP models. Serum TNF-α levels did not differ between the more severe groups which were defined by the puncture size [14]. One explanation may be the difference in pro-inflammatory response influenced by genetic background. Serum TNF-α profiles over time are different after CLP between A/J and C57BL/6J mice; therefore, we infer it could be dissimilar between rats and mice [21]. Moreover, TNF-α neutralization does not improve survival in mouse CLP model [22–24]. This underlines that serum TNF-α levels are not necessarily linked to severity of insult in this model.

Serum IL-1β levels were higher in the “5 %” and “20 %” groups at 6 h compared to the levels at 24 h. We did not observe any difference between groups at the same time or between 6 and 24 h for the “100 %” ligated group. There is little data in the literature concerning IL-1β in mouse CLP modeling. Initial descriptions of the inflammatory profile in this mouse model did not detect serum IL-1β [8]. In the mouse CLP model, this cytokine seemed to be significantly elevated in the sera of animals that died before the fifth day post-procedure [25]. In a study evaluating needle size in CLP, only the group with the largest puncture presented with a significant elevation in serum Il-1β at 24 h [13]. In our case, the lack of difference between groups may be explained by the regulation of IL-1β secretion and the small number of surviving animals in the 100 % group at 24 h [26].

Concerning serum IL-6, our model is in accordance with previous results indicating that serum IL-6 levels increase proportionally with mortality at 6 h after mouse CLP [27, 28]. As described in the literature, the 20 % and 100 % groups, which presented with the highest mortality rates, had serum IL-6 concentrations near or superior to 2000 pg/ml. This breakpoint predicts mortality within 3 days with a specificity of 97 % and sensitivity of 58 % [27]. When observing the pro- and anti-inflammatory balances, the IL-6/IL-10 ratio was higher in groups with larger lengths of ligated cecum. This ratio has been shown to be predictive of the outcome of patients with systemic inflammatory response syndrome [29–31].

We use in our model low dose of buprenorphine (0.05 mg/kg every 12 h) to achieve analgesia as stated by the Office of Laboratory Animal Welfare [32]. Morphine is known to increase pro-inflammatory mediators; however, low dose of buprenorphine seems to have no effect on mortality and inflammatory response [33, 34]. Moreover, we gave this analgesic drug both to sham and CLP mice; hence, we believe that buprenorphine is not a cofounding factor in our model.

In our study, we focused on the 20 % ligatured group. This was done since we chose this group for further experimentation and wanted to ensure that the results were consistent. Furthermore, this group corresponds to a septic shock group. The animals of this group presented both clinical and biological elements of septic shock. They were hypotensive with a MAP below 65 mmHg. The cytokine profile was in favor of a pro-inflammatory imbalance with markers predictive of mortality, such as IL-6 and the IL-6/IL-10 ratio [27, 30]. At 48 h, IL-6/IL-10 ratio increased compared to that at 24 h after CLP, which shows the persistence of inflammation. Moreover, this group presented with a drop in leucocyte count secondary to lymphopenia, which decreased even further at 24 h. Other studies described this change in a complete blood count, with the same kinetics [8, 13, 35, 36]. The lymphopenia was secondary to sepsis-induced apoptosis and is correlated to the severity of an immunosuppressive phase and its late complications [37, 38].

We acknowledge that our study has potential limitations. First, we did not perform the same analysis in the “100 %” group as we did in the “20 %” group because of the high lethality rate. We chose animals with the same genetic background, age, and gender to limit experimental variability secondary to differences in inflammatory response and maturity of the immune system [11, 39]. Because of the mice’s age, we did not have enough animals to compensate for the mortality of the 100 % ligated group. Furthermore, our study lacked evaluation of anti-inflammatory balance within the first hours post-procedure. As previous studies on mouse CLP modeling described late IL-10 serum elevation, we chose to measure levels at 24 and 48 h [13, 14]. We were not able to determine if the 100 % group had very early pro- and anti-inflammatory imbalance, which has been shown to be predictive of mortality [25]. Third, we are aware that we have not analyzed exhaustively the cytokine response to cecal ligation. For example, other cytokines such as IL-12 or interferon-γ play a central role in septic inflammatory response [40].

## Conclusions

Our study suggests that the length of cecal ligation is a major severity factor in the mouse CLP model when needle size and the number of punctures are controlled. Furthermore, it underlines differences in the inflammatory response between rats and mice. Therefore, this experimental model should be performed with high consistency in future protocol designs. In order to accurately compare studies, ligature length used in protocols should be described.
